# Fulminant development of a giant pelvic metastasis from microsatellite-stable early-onset sigmoid colon cancer with KRAS and TP53 co-mutations: a case report

**DOI:** 10.3389/fmed.2026.1900343

**Published:** 2026-08-25

**Authors:** Shouxin Wei, Sijia Yu, Yunsheng Lan

**Affiliations:** 1 Department of Gastrointestinal Surgery, Suining Central Hospital, Suining, China; 2 Department of General Practice, Suining Central Hospital, Suining, China

**Keywords:** case report, early-onset colorectal cancer, hyper-progression, interval cancer, KRAS/TP53 co-mutation

## Abstract

**Background:**

Early-onset colorectal cancer (EO-CRC) is often characterized by a highly aggressive nature and insidious onset. Herein, we report a rare case of fulminant, early-onset colon cancer presenting with a massive pelvic seeding metastasis driven by concurrent KRAS and TP53 mutations. This study aims to elucidate the molecular mechanisms underlying its hyper-progressive course and provide critical insights into clinical differential diagnosis.

**Case description:**

A 44-year-old female presented with intermittent abdominal pain for 8 months, which worsened alongside abdominal distension for 10 days prior to admission. Notably, an abdominopelvic CT scan performed 8 months earlier had yielded unremarkable findings. Upon admission, laboratory evaluations revealed a fulminant elevation of serum tumor markers (CEA: 256.63 ng/mL, CA19-9: 3,724.63 U/mL), and an abdominopelvic CT, subsequently confirmed intraoperatively, revealed a massive (~20 cm) cystic-solid pelvic mass accompanied by 1,000 mL of ascites. The primary lesion was identified as a 3.1 cm sigmoid colon adenocarcinoma. The patient successfully underwent combined radical resection. Postoperative histopathology confirmed that the pelvic mass was a metastasis originating from the colon adenocarcinoma [CDX2 (+), CK20 (+)], exhibiting lymphovascular invasion and intermediate tumor budding (Grade Bd2). Next-generation sequencing (NGS) demonstrated that the tumor was microsatellite stable (MSS) and harbored concurrent pathogenic mutations in KRAS (p.G12D, 28.70%) and TP53 (p.I195T, 42.50%), conferring intrinsic resistance to conventional anti-EGFR monoclonal antibodies and immunotherapy. Consequently, a multidisciplinary team (MDT) formulated a therapeutic strategy consisting of systemic adjuvant chemotherapy with bevacizumab plus mFOLFOX6, paired with high-frequency follow-up.

**Conclusion:**

Early-onset MSS colorectal cancer, driven by concurrent KRAS/TP53 mutations, can exhibit extreme, hyper-progressive malignant behavior. A recent negative screening result cannot entirely rule out the development of interval colorectal cancer. Clinicians must maintain a high index of suspicion for primary gastrointestinal malignancies when encountering massive cystic-solid pelvic masses in female patients. Timely multi-gene NGS panel testing is crucial to deciphering multi-drug resistant oncogenic driver axes and tailoring individualized precise treatment strategies.

## 1 Introduction

Colorectal cancer (CRC) is conventionally regarded as a relatively slow-progressing gastrointestinal malignancy, with the evolution from early adenoma to invasive carcinoma and subsequent distant metastasis typically spanning several years (1, 2). However, early-onset colorectal cancer (EO-CRC), defined as colorectal cancer diagnosed in individuals under 50 years of age, has exhibited a worrying global increase in incidence in recent years (3). Compared with late-onset cases, EO-CRC frequently exhibits heightened aggressiveness, more insidious early clinical manifestations, and distinct molecular pathological characteristics, often complicating clinical management and screening strategies (4).

Pelvic seeding or ovarian metastasis (such as Krukenberg tumor) from CRC is not uncommon in clinical practice (5). However, the fulminant hyper-progression of a lesion from an entirely negative radiologic baseline to a massive, 20-cm cystic-solid pelvic metastatic mass within a short interval represents an exceedingly rare phenomenon. This specific clinical phenotype—characterized by a “small primary tumor with large metastases” and an exceptionally short doubling time—is highly susceptible to misdiagnosis as a primary gynecological ovarian tumor (6). It simultaneously challenges conventional annual screening windows and clinical follow-up intervals, thereby increasing the risk of manifesting as clinical interval cancer (7). Although a limited number of case reports have previously described colon cancer associated with massive pelvic metastases, the vast majority are confined to macroscopic clinical presentations or routine immunohistochemical (IHC) profiles. Rare studies have elucidated the underlying deep oncogenic driver mechanisms of such hyper-progressive cases from a molecular pathological perspective (8, 9).

Herein, we report a rare case of a 44-year-old female patient with early-onset microsatellite stable (MSS) sigmoid colon adenocarcinoma who experienced a fulminant development of a 20-cm massive pelvic seeding metastatic mass within an 8-month period. This case suggests that MSS EO-CRC, when harboring concurrent KRAS/TP53 mutations alongside high-risk pathological features such as tumor budding and lymphovascular or perineural invasion, may exhibit a rapid clinical progression and a massive pelvic metastatic phenotype. These findings may provide evidence-based insights for a multidisciplinary team (MDT) to formulate individualized, precise therapeutic and follow-up strategies for managing such refractory CRC.

## 2 Case report

A 44-year-old female presented with an 8-month history of intermittent abdominal pain, which had exacerbated alongside abdominal distension for 10 days prior to admission. Her height was 160 cm and her body weight was 90 kg, corresponding to a body mass index (BMI) of 35.2 kg/m^2^. She had a 7-year history of hypertension, which was well-controlled with a combination of amlodipine besylate and enalapril maleate. Additionally, she had a 5-year history of coronary heart disease and atrioventricular nodal reentrant tachycardia (AVNRT), for which she had undergone radiofrequency catheter ablation. Notably, an abdominopelvic computed tomography (CT) scan performed at a local hospital 8 months previously had revealed no significant organic lesions. The patient had no prior history of routine colorectal cancer screening or colonoscopy evaluation before the onset of these symptoms. According to the available records from the local hospital, only the abdominopelvic CT examination was performed at that visit; serum tumor markers and endoscopic examinations were not obtained, and the detailed results of other routine laboratory tests were unavailable.

Physical examination revealed a distended abdomen with marked tenderness in the lower quadrant. A palpable mass, measuring approximately 15 cm, was noted; it was firm, poorly mobile, and tender to palpation. Admission CT demonstrated a massive cystic-solid mass with mixed density in the lower abdomen and pelvis, measuring 15.2 cm × 13.3 cm × 13.1 cm. The mass was intimately associated with the adjacent bowel and left adnexa, suggesting a potential ovarian origin. Colonoscopy revealed a space-occupying lesion in the sigmoid colon accompanied by severe luminal narrowing, which precluded the passage of the endoscope. Biopsy histopathology indicated adenocarcinoma. Laboratory evaluations showed a white blood cell count of 13.9 × 10^9^/L, a hemoglobin level of 89 g/L, and a platelet count of 710 × 10^9^/L. Serum tumor markers were significantly elevated, including carcinoembryonic antigen (CEA) at 256.63 ng/mL, carbohydrate antigen 19–9 (CA19-9) at 3,724.63 U/mL, carbohydrate antigen 125 (CA125) at 420.72 U/mL, and human epididymis protein 4 (HE4) at 183.8 pmol/L. The Risk of Ovarian Malignancy Algorithm (ROMA) scores for premenopausal and postmenopausal status were 68.7 and 85.3%, respectively.

Following a multidisciplinary team (MDT) discussion and thorough preoperative preparation, the patient underwent exploratory laparotomy 10 days after admission. Intraoperatively, approximately 1,000 mL of straw-colored ascites was observed. The pelvic mass had further enlarged compared with the preoperative imaging findings, presenting as a massive, approximately 20-cm cystic-solid tumor, which exhibited severe adhesion and indistinct planes with the sigmoid colon, left ovary, and portions of the left pelvic wall. Consequently, sigmoidectomy, excision of the massive pelvic tumor, left adnexectomy, and partial pelvic peritonectomy were performed. Postoperatively, supportive treatments were administered, including anti-infective therapy, correction of anemia, and nutritional support. The patient recovered uneventfully and was discharged in stable condition on postoperative day 15.

Postoperative gross examination revealed an ulcerative tumor (3.1 cm × 2.2 cm × 1.6 cm) in the sigmoid colon with circumferential growth; the pelvic cavity contained a massive nodular, cystic-solid mass (19.2 cm × 17.5 cm × 9.1 cm) filled with clear fluid, which had entirely replaced the left ovarian tissue. Histopathological analysis established a diagnosis of moderately differentiated ulcerative adenocarcinoma of the sigmoid colon, invading the subserosal layer, with accompanied perineural and lymphovascular invasion, as well as intermediate-grade tumor budding (Grade Bd2). IHC analysis of the pelvic mass demonstrated positivity for CDX2, SATB2, and CK20, but negativity for CK7 and Pax-8, confirming that the massive pelvic mass originated from the sigmoid colon adenocarcinoma metastasis. Furthermore, the mismatch repair (MMR) proteins (MSH2, MSH6, MLH1, and PMS2) all exhibited proficient (positive) expression; p53 immunostaining exhibited a mutant-type pattern, and the Ki-67 proliferation index was approximately 70%. Next-generation sequencing (NGS) confirmed a microsatellite stable (MSS) phenotype, harboring a missense mutation in KRAS (p.G12D, variant allele frequency [VAF]: 28.70%) and a missense mutation in TP53 (p.I195T, VAF: 42.50%). Additionally, missense mutations were detected in FGFR1 (VAF: 67.31%), SETD2 (VAF: 56.85%), CDK12 (VAF: 41.66%), and MLH3 (VAF: 40.52%).

**Figure 1:**
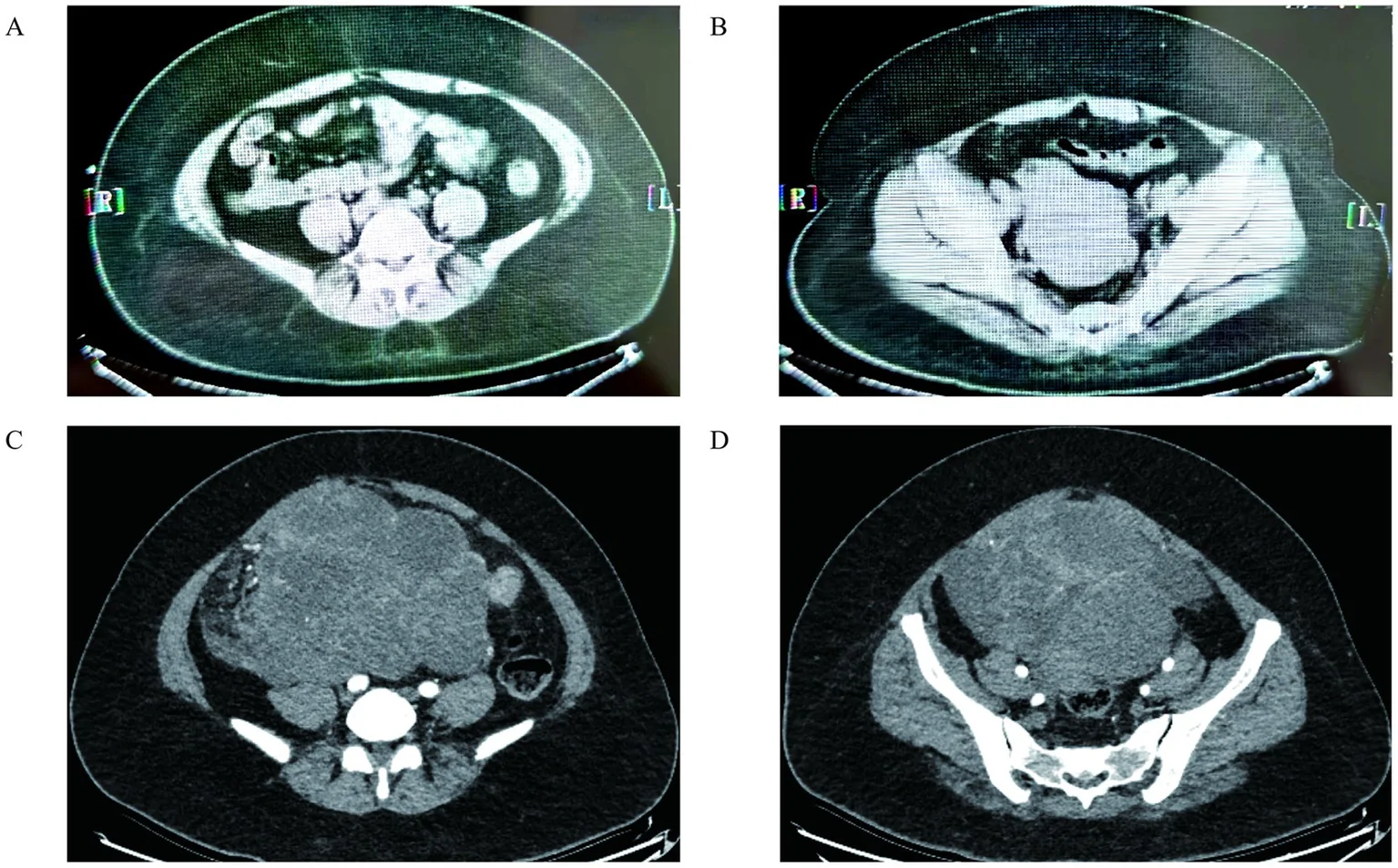
Comparison of abdominopelvic computed tomography (CT) scans between 8 months ago and admission, revealing a hyper-progressive pelvic giant metastasis **(A,B)** Axial abdominopelvic CT scans performed at a local hospital 8 months ago, showing no obvious organic or space-occupying lesions within the abdominal and pelvic cavities, serving as baseline normal imaging. **(C,D)** Axial contrast-enhanced pelvic CT scans obtained upon the current admission, demonstrating a massive, mixed density, mixed solid-cystic cystic-solid neoplastic lesion in the lower abdomen and pelvic cavity, measuring approximately 15.2 cm × 13.3 cm × 13.1 cm. The giant pelvic mass is closely adjacent to the adjacent bowel loops and the left adnexa, mimicking a primary gynecological/ovarian tumor.

**Figure 2:**
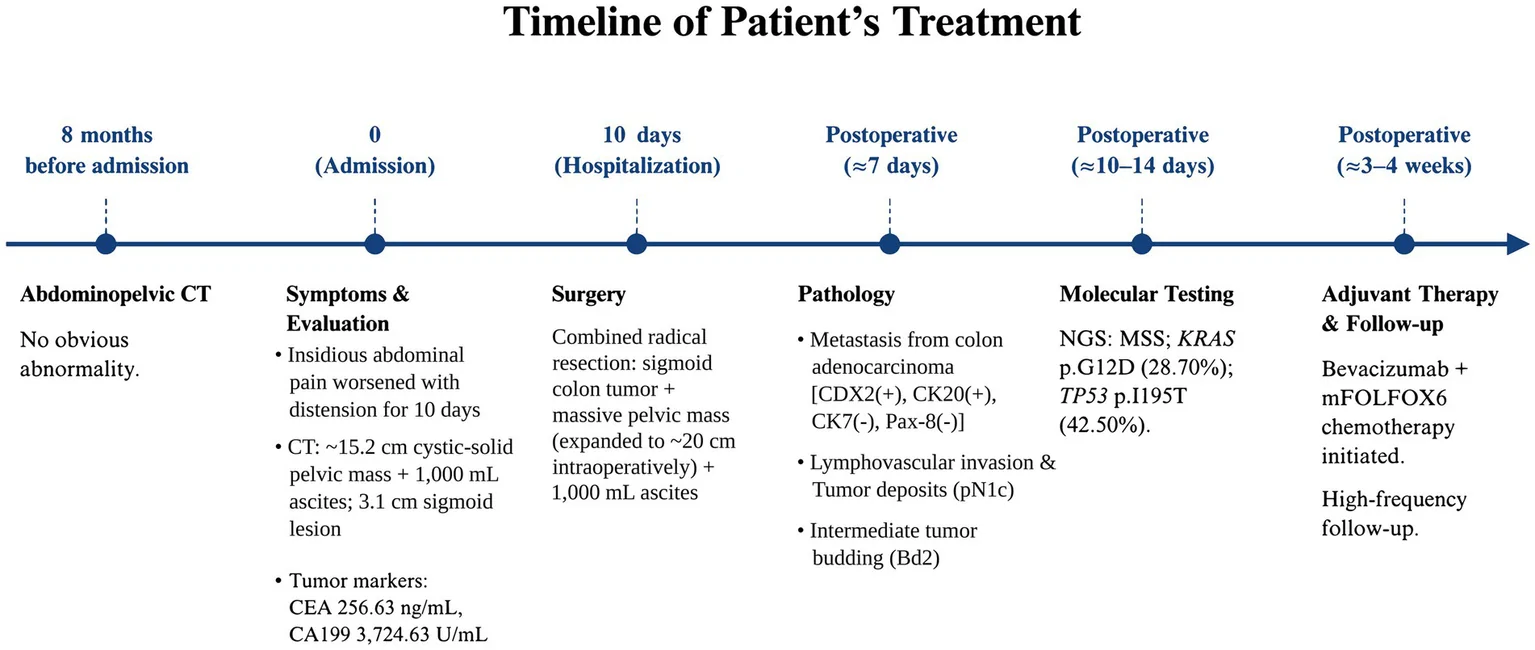
Timeline of the clinical course, diagnostic evaluations, surgical interventions, and postoperative management of the patient.

**Figure 3:**
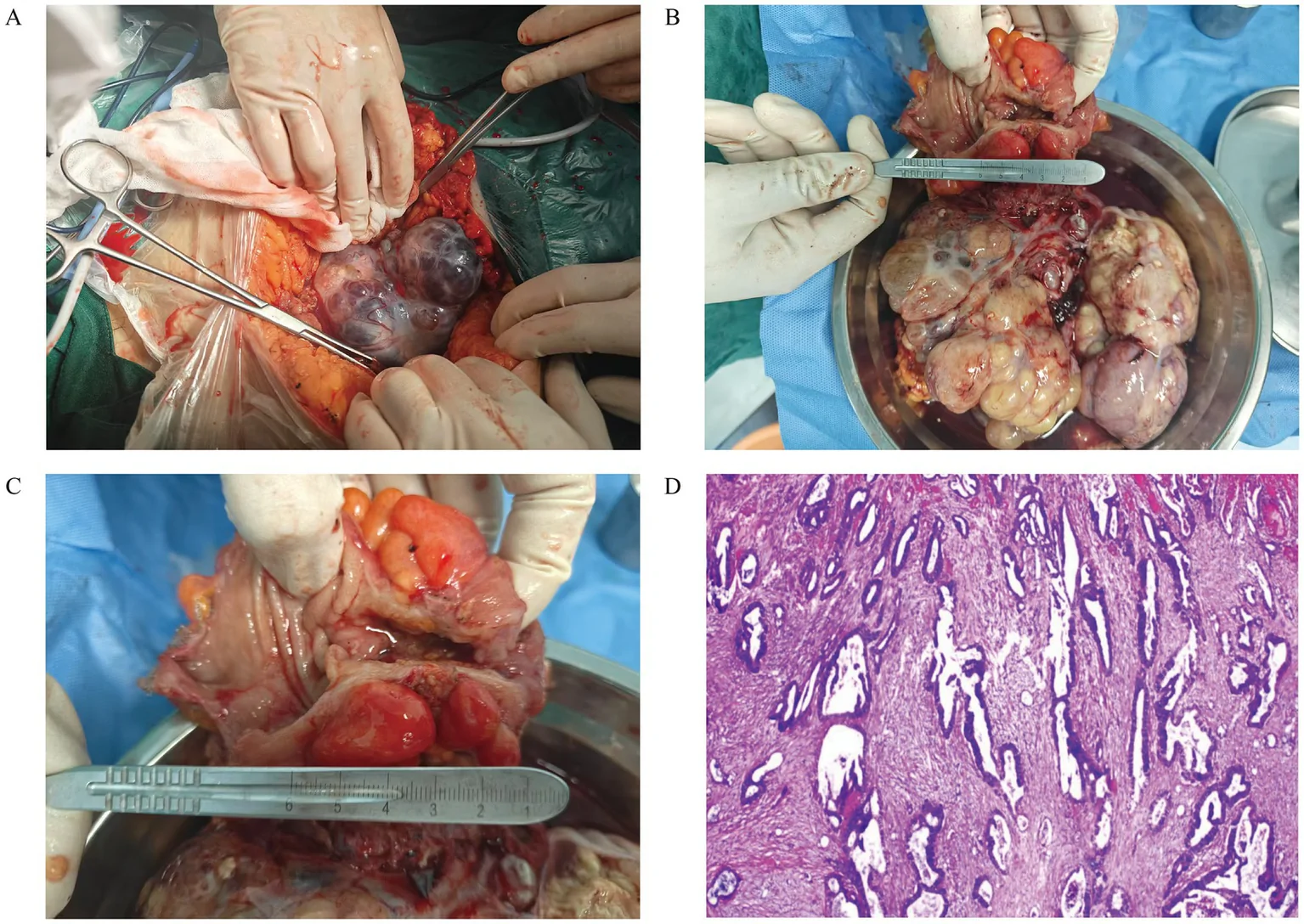
Intraoperative and pathological findings of the primary tumor and pelvic mass **(A)** Intraoperative photograph showing a giant, nodular, cystic-solid pelvic mass. **(B)** Macroscopic view of the resected sigmoid colon tumor and the giant pelvic mass. **(C)** Close-up view of the bisected sigmoid colon, revealing an ulcerative primary tumor. **(D)** Histopathology of the primary tumor confirming moderately differentiated adenocarcinoma infiltrating the subserosa (H&E staining, 100×).

Given that the presence of the KRAS p.G12D mutation confers intrinsic resistance to conventional anti-EGFR monoclonal antibodies, the MDT formulated a systemic postoperative adjuvant chemotherapy regimen consisting of mFOLFOX6 combined with bevacizumab. Currently, the patient remains under close postoperative follow-up (Figures 1–3).

## 3 Discussion

This report describes a rare case of EO-CRC presenting with a fulminant malignant progression, here, ‘rapid’ refers not merely to subjectively fast symptom onset, but to objective radiological and biological hyper-progression: wherein the patient rapidly developed a massive, nearly 20-cm pelvic seeding metastatic mass despite having an entirely negative CT baseline examination merely 8 months prior. Clinically, pelvic seeding metastases derived from the gastrointestinal tract are frequently misdiagnosed as primary gynecological ovarian tumors due to their radiographic overlap and massive cystic-solid presentations (9, 10). In this case, preoperative serum tumor markers were remarkably elevated (CEA: 256.63 ng/mL, CA19-9: 3,724.63 U/mL, CA125: 420.72 U/mL), and the apparent total replacement of the left ovary by the mass created a highly deceptive clinical scenario. Ultimately, the definitive diagnosis was established following surgical resection, with IHC profiling confirming that the pelvic mass exhibited a classic gastrointestinal phenotype. Furthermore, NGS was utilized to decipher the underlying deep molecular biological mechanisms driving this hyper-progressive malignant evolution (Table 1).

**Table 1:** Clinical characteristics, laboratory findings, immunohistochemical profiling, and genomic mutations of the patient.

Parameter/biomarker	Value/genomic status	Reference range/clinical significance
Baseline characteristics		
Age/gender	44 / Female	—
BMI	35.2 kg/m2	Obesity
Interval since normal CT	8 months	Hyper-progressive/interval cancer
Laboratory findings		
White blood cell	13.9 × 109/L	(3.5–9.5) x 109/L (Elevated)
Platelet	710 × 109/L	(100–350) x 109/L (Severe thrombocytosis)
Hemoglobin	89 g/L	(115–150) g/L (Moderate anemia)
CEA	256.63 ng/mL	< 5.0 ng/mL (Markedly elevated)
CA19-9	3724.63 U/mL	< 37.0 U/mL (Markedly elevated)
CA125	420.72 U/mL	< 35.0 U/mL (Elevated)
HE4	183.8 pmol/L	< 70.0 pmol/L (Elevated)
ROMA index (Pre- /post-menopausal)	68.7% / 85.3%	High risk for malignancy
Immunohistochemical profiling		
pTNM	pT3N1cM1c	IVC
CDX2 (Primary and pelvic mass)	Positive (+)	Confirmed colorectal differentiation
SATB2 (Pelvic mass)	Positive (+)	Confirmed colorectal differentiation
CK20 (Pelvic mass)	Positive (+)	Confirmed colorectal differentiation
CK7 (Pelvic mass)	Negative (−)	Excluded primary gynecological malignancy
Pax-8 (Pelvic mass)	Negative (−)	Excluded primary gynecological malignancy
MSH2/MSH6/MLH1/PMS2	Positive (+, intact)	Proficient mismatch repair status
p53	Positive (+, mutant pattern)	Aberrant p53 overexpression (Tumor suppressor loss)
Ki-67	~70%	High cellular proliferation rate
NGS molecular profiling		
Microsatellite status	MSS	Microsatellite stable
*KRAS* mutation	p.G12D, 28.70%	Oncogene activation; Resistance to anti-EGFR therapies
*TP53* mutation	p.I195T, 42.50%	Loss of transactivation (Consistent with mutant p53 IHC)
*FGFR1* variant	p.L7R, 67.31%	Class III variant
*SETD2* variant	p.G901R, 56.85%	Class III variant
*CDK12* variant	p.R112C, 41.66%	Class III variant
*MLH3* variant	p.I988M, 40.52%	Class III variant

BMI, body mass index; CT, computed tomography; CEA, carcinoembryonic antigen; CA19-9, carbohydrate antigen 19–9; CA125, carbohydrate antigen 125; HE4, human epididymis protein 4; ROMA, ovarian malignancy risk algorithm; pTNM, pathological tumor-node-metastasis staging based on the American Joint Committee on Cancer (AJCC) 8th edition; IHC, immunohistochemistry; pMMR, proficient mismatch repair; NGS, next-generation sequencing; MSS, microsatellite stable; EGFR, epidermal growth factor receptor.

This patient exhibited a characteristic phenotype consisting of a small primary tumor, extensive distant metastases, and an exceptionally short tumor doubling time. The presence of intermediate-grade tumor budding (Grade Bd2) identified via histopathology provided a morphological rationale for this clinical phenomenon. Tumor budding is widely recognized as a microscopic hallmark of the epithelial-mesenchymal transition (EMT) in CRC, indicating that the carcinoma cells had acquired heightened migratory, invasive, and basement membrane-penetrating capacities. This served as the pathological foundation for the extensive peritoneal seeding and the 20-cm massive pelvic lesion, despite the primary sigmoid lesion measuring only 3.1 cm (11, 12). Genomic profiling further elucidated the causes of this rapid progression; specifically, the concurrent KRAS and TP53 co-mutations formed a molecular storm driving this hyper-progressive course. In the traditional Vogelstein model of CRC carcinogenesis, KRAS activation and TP53 inactivation typically occur as sequential, multi-step events (13, 14). However, translational research has demonstrated that concurrent mutations in both genes exert a potent synergistic empowering effect (15). The KRAS p.G12D mutation, located within the GTP-binding domain, leads to constitutive activation of the downstream MAPK pathway, providing sustained signals for unconstrained cell proliferation. Concurrently, the TP53 missense mutation within the DNA-binding domain results in the loss of transactivation activity of the encoded p53 protein. Consequently, it fails to initiate cell cycle arrest or apoptosis to counteract this aberrant proliferation, enabling tumor cells to evade apoptosis and undergo aggressive, uninhibited growth (16, 17). Furthermore, alongside the aforementioned co-mutations, this case presented with missense mutations in CDK12 and SETD2. Although classified as variants of uncertain significance (VUS, Class III), functional genomics reveals that CDK12 regulates the transcription of genes involved in homologous recombination repair of DNA double-strand breaks, whereas SETD2, encoding a histone methyltransferase, is indispensable for the fidelity of DNA mismatch repair (18, 19). Point mutations in these two genes may potentially aggravate genomic instability, thereby accelerating malignant progression under the synergistic influence of the KRAS/TP53 axis (Table 2).

**Table 2:** Systematic comparison of published colorectal cancer cases with giant ovarian or pelvic metastases.

Study	Age; primary site; timing	Metastatic lesion	Presentation and tumor markers	Diagnostic features	Treatment and reported outcome
Ongom et al. (6)	58; ascending colon; metachronous (~3 years)	Bilateral ovarian tumors, 30 and 28 cm; peritoneal and cutaneous metastases	Abdominal distension and massive ascites; serum markers not reported	CK7 (−), CK20 (+), CDX2 (+); abnormal MMR/MSI profile suggesting probable Lynch syndrome or sporadic MSI-H	Bilateral salpingo-oophorectomy and systemic chemotherapy; outcome not reported
Tajima et al. (22)	47; sigmoid colon; synchronous	Bilateral ovarian metastases; dominant tumor 18 × 15 × 11 cm; limited peritoneal disease	Distension, massive ascites, bilateral pleural effusions; CEA 335.2 ng/mL; CA125 219 U/mL	CK20 (+), CK7 (−); moderately differentiated adenocarcinoma	Sigmoidectomy, bilateral oophorectomy, hysterectomy, omentectomy, FOLFOX; NED at 78 months after later liver resection
Zogbi et al. (23)	57; sigmoid colon; metachronous (6 years)	Unilateral ovarian metastasis, 30 × 28.5 × 14 cm; 10 L cystic content	Abdominal pain and giant palpable mass; CEA 2,100 ng/mL; CA125 87.4 U/mL	CK20 (+), CDX2 (+), CEA (+), CK7 (−)	Complete surgical excision; uncomplicated early postoperative recovery; long-term outcome not reported
Lam and Ong (24)	51; cecum; metachronous (12 months)	Right ovarian metastasis, 23 × 21 × 20 cm; limited peritoneal nodules	Abdominal discomfort; rising CEA 228 ng/mL; progression during five cycles of FOLFOX	Biopsy and resection confirmed metastatic mucinous adenocarcinoma of colorectal origin	R0 ovarian/peritoneal resection and prophylactic contralateral salpingo-oophorectomy; no recurrence at 6 months
Gaur et al. (25)	35; rectum; synchronous (known for 1 year)	Bilateral ovarian metastases, 20 × 15 cm and 18 × 15 cm; peritoneal, omental, and liver deposits	Intestinal obstruction, pain, distension, rectal bleeding; CA125 276 U/mL	CK20 (+), CDX2 (+), CK7 (−); signet-ring cells in mucin pools	Palliative bilateral salpingo-oophorectomy and diversion colostomy, followed by palliative chemotherapy
Maeda et al. (7)	58; cecum; synchronous	Ovarian metastasis, 34 × 28 cm	Distension and lower abdominal pain; CEA 142 ng/mL; CA19-9117 U/mL; CA125 328 U/mL	CK7 (−), CK20 (+), CDX2 (+), ER (−); initially misdiagnosed as primary ovarian cancer	Ovariectomy and right colectomy after ineffective ovarian-cancer chemotherapy; outcome not reported
Present case	44; sigmoid colon; synchronous/interval presentation within 8 months	Pelvic/left ovarian metastasis, 19.2 × 17.5 × 9.1 cm; ascites and peritoneal involvement	Pain and rapidly progressive distension; CEA 256.63 ng/mL; CA19-9 3,724.63 U/mL; CA125 420.72 U/mL	CDX2 (+), SATB2 (+), CK20 (+), CK7 (−), Pax-8 (−); MSS; KRAS p.G12D and TP53 p.I195T	Combined radical resection followed by bevacizumab plus mFOLFOX6; ongoing close follow-up

CEA, carcinoembryonic antigen; CA125, carbohydrate antigen 125; CA19-9, carbohydrate antigen 19–9; MMR, mismatch repair; MSI-H, microsatellite instability-high; MSS, microsatellite stable; ER, estrogen receptor; FOLFOX, folinic acid, fluorouracil, and oxaliplatin; NED, no evidence of disease.

From a prognostic perspective, this case presents with multiple high-risk factors. KRAS-mutant CRC is conventionally associated with a poor prognosis, intrinsic resistance to anti-EGFR therapy, and limited therapeutic options (20). Nevertheless, the landscape of KRAS-targeted therapies is rapidly evolving. Currently approved KRAS inhibitors for CRC primarily target the KRAS p.G12C mutation, whereas inhibitors and combination strategies targeting the p.G12D alteration remain predominantly in clinical trial phases (21). Moving forward, close follow-up based on standardized chemotherapy and anti-angiogenic therapy is imperative, alongside the dynamic monitoring of CEA, CA19-9, and CA125 levels, as well as radiographic alterations. In the event of disease recurrence or progression, second-line treatments, locoregional therapies, and eligibility for relevant clinical trials should be evaluated, taking into account the patient’s prior treatment response, performance status, and molecular profiling.

### 3.1 Patient perspective

The patient initially experienced profound shock and anxiety regarding her diagnosis of advanced colon cancer, which presented as sudden abdominal distension despite completely normal screening findings merely 8 months prior. Following efficient surgical resection managed by the MDT, the compressive symptoms caused by the massive pelvic tumor were significantly alleviated. This clinical improvement effectively restored the patient’s confidence in confronting her illness. Currently, she maintains a resilient and collaborative outlook, adhering closely to the postoperative systemic adjuvant chemotherapy regimen and the scheduled clinical follow-up protocol.

## Data Availability

The original contributions presented in the study are included in the article/supplementary material, further inquiries can be directed to the corresponding author.

## References

[1] Smit WL Spaan CN de Boer RJ Ramesh P Martins Garcia T Meijer BJ . Driver mutations of the adenoma-carcinoma sequence govern the intestinal epithelial global translational capacity. Proc Natl Acad Sci USA. (2020) 117:25560–70. doi: 10.1073/pnas.1912772117, 32989144 PMC7568276

[2] Jones S Chen WD Parmigiani G Diehl F Beerenwinkel N Antal T . Comparative lesion sequencing provides insights into tumor evolution. Proc Natl Acad Sci USA. (2008) 105:4283–8. doi: 10.1073/pnas.0712345105, 18337506 PMC2393770

[3] Sung H Siegel RL Laversanne M Jiang C Morgan E Zahwe M . Colorectal cancer incidence trends in younger versus older adults: an analysis of population-based cancer registry data. Lancet Oncol. (2025) 26:51–63. doi: 10.1016/S1470-2045(24)00600-4, 39674189 PMC11695264

[4] Lawler T Parlato L Warren Andersen S. The histological and molecular characteristics of early-onset colorectal cancer: a systematic review and meta-analysis. Front Oncol. (2024) 14:1349572. doi: 10.3389/fonc.2024.1349572, 38737895 PMC11082351

[5] Shi J Huang A Song C Li P Yang Y Gao Z . Effect of metastasectomy on the outcome of patients with ovarian metastasis of colorectal cancer: a systematic review and meta-analysis. Eur J Surgical Oncol. (2023) 49:106961. doi: 10.1016/j.ejso.2023.06.013, 37355393

[6] Ongom PA Odida M Lukande RL Jombwe J Elobu E. Metastatic colorectal carcinoma mimicking primary ovarian carcinoma presenting as 'giant' ovarian tumors in an individual with probable lynch syndrome: a case report. J Med Case Rep. (2013) 7:158. doi: 10.1186/1752-1947-7-158, 23787146 PMC3694008

[7] Maeda Y Minagawa N Shoji H Kobayashi T Yamamoto K. Giant ovarian tumor with colorectal cancer: suggestion concerning the need for colonoscopy screening in cases with large ovarian tumor-a report of three cases. BMC Surg. (2022) 22:111. doi: 10.1186/s12893-022-01565-4, 35321695 PMC8943954

[8] Sugarbaker PH Liang J. Ovarian metastases from right colon cancer treated with systemic cancer chemotherapy, a case report. Int J Surg Case Rep. (2018) 47:25–9. doi: 10.1016/j.ijscr.2018.04.017, 29705675 PMC5994710

[9] Dundr P Singh N Nožičková B Němejcová K Bártů M Stružinská I. Primary mucinous ovarian tumors vs. ovarian metastases from gastrointestinal tract, pancreas and biliary tree: a review of current problematics. Diagn Pathol. (2021) 16:20. doi: 10.1186/s13000-021-01079-2, 33706757 PMC7953678

[10] Zulfiqar M Koen J Nougaret S Bolan C VanBuren W McGettigan M . Krukenberg Tumors: update on imaging and clinical features. AJR Am J Roentgenol. (2020) 215:1020–9. doi: 10.2214/AJR.19.22184, 32755184

[11] Nie F Sun X Sun J Zhang J Wang Y. Epithelial-mesenchymal transition in colorectal cancer metastasis and progression: molecular mechanisms and therapeutic strategies. Cell Death Discovery. (2025) 11:336. doi: 10.1038/s41420-025-02593-8, 40695791 PMC12284022

[12] Maffeis V Nicolè L Cappellesso R. RAS, cellular plasticity, and tumor budding in colorectal Cancer. Front Oncol. (2019) 9:1255. doi: 10.3389/fonc.2019.01255, 31803624 PMC6877753

[13] Fearon ER Vogelstein B. A genetic model for colorectal tumorigenesis. Cell. (1990) 61:759–67. doi: 10.1016/0092-8674(90)90186-I, 2188735

[14] Yang L Wang S Lee JJ Lee S Lee E Shinbrot E . An enhanced genetic model of colorectal cancer progression history. Genome Biol. (2019) 20:168. doi: 10.1186/s13059-019-1782-4, 31416464 PMC6694562

[15] Tang Y Fan Y. Combined KRAS and TP53 mutation in patients with colorectal cancer enhance chemoresistance to promote postoperative recurrence and metastasis. BMC Cancer. (2024) 24:1155. doi: 10.1186/s12885-024-12776-8, 39289671 PMC11409552

[16] Navarro-Jiménez M González B Mulet N Hierro C Alonso S. KRAS-targeted therapies in colorectal cancer: a systematic analysis of mutations, inhibitors, and clinical trials. NPJ Precision Oncology. (2025) 9:380. doi: 10.1038/s41698-025-01166-3, 41299036 PMC12658183

[17] Wang H Guo M Wei H Chen Y. Targeting p53 pathways: mechanisms, structures, and advances in therapy. Signal Transduct Target Ther. (2023) 8:92. doi: 10.1038/s41392-023-01347-1, 36859359 PMC9977964

[18] Blazek D Kohoutek J Bartholomeeusen K Johansen E Hulinkova P Luo Z . The cyclin K/Cdk12 complex maintains genomic stability via regulation of expression of DNA damage response genes. Genes Dev. (2011) 25:2158–72. doi: 10.1101/gad.16962311, 22012619 PMC3205586

[19] Li F Mao G Tong D Huang J Gu L Yang W . The histone mark H3K36me3 regulates human DNA mismatch repair through its interaction with MutSα. Cell. (2013) 153:590–600. doi: 10.1016/j.cell.2013.03.025, 23622243 PMC3641580

[20] Takeda M Yoshida S Inoue T Sekido Y Hata T Hamabe A . The role of KRAS mutations in colorectal Cancer: biological insights, clinical implications, and future therapeutic perspectives. Cancer. (2025) 17:428. doi: 10.3390/cancers17030428, 39941797 PMC11816235

[21] Shi K Li A. Targeting KRAS G12D: advances in inhibitor design. Thoracic Cancer. (2025) 16:e70203. doi: 10.1111/1759-7714.70203, 41423807 PMC12719395

[22] Tajima Y Kameyama H Yamada S Yagi R Nakano M Nagahashi M . Long-term survival in pseudo-Meigs' syndrome caused by ovarian metastases from colon cancer. World J Surg Oncol. (2016) 14:286. doi: 10.1186/s12957-016-1040-0, 27842595 PMC5109729

[23] Zogbi L Isaías A Machado PA Neutzling A Juliano C. Krukenberg's tumour unilateral giant metachronous of colonic origin - case report. Int J Surg Case Rep. (2017) 41:184–7. doi: 10.1016/j.ijscr.2017.10.029, 29096339 PMC5686220

[24] Lam D Ong E. The role of surgical excision for the Krukenberg tumour: a case report. Int J Surg Case Rep. (2017) 38:185–8. doi: 10.1016/j.ijscr.2017.07.046, 28772202 PMC5540708

[25] Gaur NK Shaikh O Vijayakumar C Kumbhar U Nachiappa Ganesh R. Intestinal obstruction due to giant bilateral Krukenberg's tumors synchronous of colorectal origin. Cureus. (2021) 13:e14176. doi: 10.7759/cureus.14176, 33936887 PMC8080991

